# Supplementary material for: Knowledge, attitude and practice of healthcare providers on mistreatment of women during labour and childbirth: A cross-sectional study in Tehran, Iran, 2021
Source: PLoS One. 2024 Oct 3;19(10):e0311346. doi: 10.1371/journal.pone.0311346 (PMC11449288; doi:10.1371/journal.pone.0311346)
Supplement: S1 Appendix — (DOCX) [file pone.0311346.s001.docx]

**S1 Appendix. Characteristics and names of study hospitals, 2021 data.**

| **Characteristics** | **Hospitals (Geographical location in Tehran/ name)** | | | | |
| --- | --- | --- | --- | --- | --- |
|  | **North/ Taleghani** | **South/ Mahdieh** | **East/ Arash** | **West/ Rasoul Akram** | **Center/ Valiasr** |
| **Health outcomes** |  |  |  |  |  |
| Total births | 641 | 5630 | 5332 | 347 | 2140 |
| Number of vaginal births | 277 | 2533 | 1894 | 105 | 454 |
| Number of caesarean births | 364 | 3097 | 3438 | 242 | 1686 |
| Number of live births | 655 | 5696 | 5430 | 359 | 2208 |
| Stillbirths | 2 | 111 | 77 | 6 | 45 |
| Maternal deaths | 4 | 0 | 0 | 9 | 15 |
| **Staffing** |  |  |  |  |  |
| Number of obstetricians | 8 | 10 | 8 | 7 | 18 |
| Number of midwives | 18 | 34 | 26 | 11 | 39 |
| Number of residents | 15 | 36 | 30 | 21 | 31 |
| **Capacity** |  |  |  |  |  |
| Number of labour and delivery room | 3 | 10 | 9 | 3 | 1 |
| Number of beds in labour and delivery rooms | 4 | 10 | 10 | 3 | 1 |
| Designated waiting room for family members or companions | 0 | 0 | 0 | 0 | 0 |

Resource: Department of Midwifery, Ministry of Health and Medical Education (MOHME), Iran; 2021
