# Supplementary material for: Knowledge, attitude and practice of healthcare providers on mistreatment of women during labour and childbirth: A cross-sectional study in Tehran, Iran, 2021
Source: PLoS One. 2024 Oct 3;19(10):e0311346. doi: 10.1371/journal.pone.0311346 (PMC11449288; doi:10.1371/journal.pone.0311346)
Supplement: S2 Appendix — (DOCX) [file pone.0311346.s002.docx]

**S2 Appendix. Questionnaire**

1. **Socio-demographic characteristics**

| **No.** | **Question** | **Possible option** |
| --- | --- | --- |
| 1 | Name of hospital |  |
| 2 | How old are you? |  |
| 3 | What is your marital status? | A. Single  B. Married  C. Divorced  D. Widowed |
| 4 | What is your profession? | A. Student (Go to Question 5)  B. Midwife  C. Obstetrician |
| 5 | What is your field of study? | A. Midwifery  B. Medical intern  C. First year resident  D. Second year resident  E. Third year resident  F. Fourth year resident |
| 6 | What best describes your monthly income (IRR)? (Please check one) | A. No income  B. 10 000 000-50 000 000  C. 60 000 000-100 000 000  D. 110 000 000-150 000 000  E. More than 150 000 000 |
| 7 | How many years do you have work experience? |  |
| 8 | How many night shifts do you have per month? |  |
| 9 | Have you ever been trained on respectful maternity care or maternity mistreatment? | A. Yes (Go to Question 10)  B. No |
| 10 | What kind of training was it? |  |

1. **Knowledge items**

*Please read the questions carefully and choose the appropriate option.*

| **No.** | **Item** | **Yes** | **NO** | **Do not know** |
| --- | --- | --- | --- | --- |
| 1 | In your opinion, can putting pressure on women’s abdomens during childbirth (applying fundal pressure) be considered as mistreatment? |  |  |  |
| 2 | In your opinion, is using the phrase “If you do not give birth, you will be taken to the operating room for a cesarean section” a form of mistreatment? |  |  |  |
| 3 | A woman states that she has been waiting in labour for a long time to receive services (in her opinion, for no reason). In your opinion, there has been disrespectful care? |  |  |  |
| 4 | A woman tends to have a squatting position at birth, and this permission has not been given to her. In your opinion, has there been disrespectful care? |  |  |  |
| 5 | One woman stated that she had been asked for rewards for the birth of her baby. In your opinion, has there been disrespectful care? |  |  |  |
| 6 | In labour, women do not collaborate, and you force her by saying, “If you do not collaborate and a problem arises for you or your baby, you are responsible for the consequences”. In your opinion, has there been disrespectful care? |  |  |  |
| 7 | Despite her consent, a woman was examined by maternity healthcare providers for various reasons during labour and delivery. In your opinion, is this a form of mistreatment? |  |  |  |
| 8 | In labour, vaginal examinations are performed without using curtains or partitions, and in the presence of other labouring women. In your opinion, is this a form of mistreatment? |  |  |  |
| 9 | In labour, a woman screams in pain and you invite her to be silent with your finger. In your opinion, has there been disrespectful care? |  |  |  |
| 10 | The labouring woman was asked not to get out of bed and not walk while in the labour ward. In your opinion, is this a form of mistreatment? |  |  |  |

11. The labouring woman states that she has received fewer services because of her condition (e.g., HIV or addiction). What kind of mistreatment do you think has occurred?

A. Lack of supportive care

B. Neglect and abandonment

C. Discriminatory care

D. Do not know

1. **Attitude items**

*Please select the number according to your opinion.*

1 = strongly agree, 2 = agree, 3 = uncertain, 4 = disagree, 5 = strongly disagree

| **No.** | **Item** | **1** | **2** | **3** | **4** | **5** |
| --- | --- | --- | --- | --- | --- | --- |
| 1 | Sometimes, physical contact (slapping the thighs) can be used to encourage pregnant women to collaborate. |  |  |  |  |  |
| 2 | Sometimes, it is necessary for healthcare providers to force a labouring woman to collaborate with subtle threats. |  |  |  |  |  |
| 3 | It is not always possible to provide equal services to all women during labour and delivery. |  |  |  |  |  |
| 4 | During birth, a labouring woman’s husband or companion should not be present in the delivery room. |  |  |  |  |  |
| 5 | Each vaginal examination should be explained to the woman, and her consent should be sought. |  |  |  |  |  |
| 6 | If necessary, physical contact (such as fundal pressure) can be used to speed up the delivery process. |  |  |  |  |  |
| 7 | Sometimes, shouting at the labouring woman can calm her down and get her to collaborate better. |  |  |  |  |  |
| 8 | Sometimes, when the number of labouring women is high, it is right to be less present at their bedside. |  |  |  |  |  |
| 9 | Sometimes, it is not necessary to explain all the information to women who have a lower level of education and may not realize them or be confused. |  |  |  |  |  |
| 10 | Labouring women should not be allowed to consume food or fluids during labour. |  |  |  |  |  |
| 11 | It is not always necessary to inform the companion of labouring women about the progress of labour and delivery. |  |  |  |  |  |
| 12 | It is alright if it is not possible to create privacy for women to be examined in the labour room. |  |  |  |  |  |
| 13 | There is not enough staff in the hospital to provide quality care to labouring women. Therefore, it is common to reduce the quality of the services provided. |  |  |  |  |  |

1. **Practice items**

*Please select the number according to your opinion.*

1 = always, 2 = often, 3 = sometimes, 4 = rarely, 5 = never

| **No.** | **Item** | **1** | **2** | **3** | **4** | **5** |
| --- | --- | --- | --- | --- | --- | --- |
| 1 | I welcome labouring woman warmly. |  |  |  |  |  |
| 2 | I introduce myself to labouring woman. |  |  |  |  |  |
| 3 | I show labouring woman around the labour unit. |  |  |  |  |  |
| 4 | I establish friendly and appropriate relationship with labouring woman. |  |  |  |  |  |
| 5 | I provide a comfortable environment for labouring woman. |  |  |  |  |  |
| 6 | I keep medical records and the results of examinations and consultations confidential. |  |  |  |  |  |
| 7 | I respect beliefs and culture of labouring woman and her companions. |  |  |  |  |  |
| 8 | I talk to labouring woman about the pain and how to relieve it. |  |  |  |  |  |
| 9 | I encourage labouring woman to call me if needed. |  |  |  |  |  |
| 10 | I pay attention to labouring woman’s safety in providing care and interventions. |  |  |  |  |  |
| 11 | I cover labouring woman’s body during examinations, using sheets. |  |  |  |  |  |
| 12 | I may slap labouring woman’s thighs to encourage her to collaborate at birth. |  |  |  |  |  |
| 13 | I may be harsher with her if a woman does not collaborate. |  |  |  |  |  |
| 14 | I may apply pressure on a woman’s abdomen (fundal pressure) to speed up the birth process. |  |  |  |  |  |
