# Supplementary material for: Knowledge, attitude and practice of healthcare providers on mistreatment of women during labour and childbirth: A cross-sectional study in Tehran, Iran, 2021
Source: PLoS One. 2024 Oct 3;19(10):e0311346. doi: 10.1371/journal.pone.0311346 (PMC11449288; doi:10.1371/journal.pone.0311346)
Supplement: S3 Appendix — (DOCX) [file pone.0311346.s003.docx]

**S3 Appendix. Content validity of the questionnaire**

1. **Knowledge items**

| **Item** | **Content Validity Index (CVI)** | | **Content Validity Ratio (CVR)** |
| --- | --- | --- | --- |
|  | I-CVI (Relevance) | S-CVI |  |
| 1 | 0.90 | 0.91 | 0.80 |
| 2 | 0.90 |  | 0.80 |
| 3 | 0.80 |  | 0.80 |
| 4 | 0.90 |  | 0.80 |
| 5 | 0.80 |  | 0.80 |
| 6 | 1.0 |  | 0.80 |
| 7 | 1.0 |  | 0.80 |
| 8 | 1.0 |  | 1.0 |
| 9 | 0.90 |  | 0.80 |
| 10 | 0.90 |  | 1.0 |
| 11 | 1.0 |  | 0.80 |

1. **Attitude items**

| **Item** | **Content Validity Index (CVI)** | | **Content Validity Ratio (CVR)** |
| --- | --- | --- | --- |
|  | I-CVI (Relevance) | S-CVI |  |
| 1 | 1.0 | 0.90 | 0.80 |
| 2 | 0.90 |  | 0.80 |
| 3 | 0.90 |  | 0.80 |
| 4 | 0.80 |  | 0.80 |
| 5 | 0.90 |  | 0.80 |
| 6 | 0.90 |  | 0.80 |
| 7 | 0.90 |  | 0.80 |
| 8 | 0.90 |  | 0.80 |
| 9 | 0.80 |  | 0.80 |
| 10 | 0.80 |  | 0.80 |
| 11 | 1.0 |  | 0.80 |
| 12 | 0.90 |  | 0.80 |
| 13 | 1.0 |  | 1.0 |

1. **Practice items**

| **Item** | **Content Validity Index (CVI)** | | **Content Validity Ratio (CVR)** |
| --- | --- | --- | --- |
|  | I-CVI (Relevance) | S-CVI |  |
| 1 | 1.0 | 0.97 | 1.0 |
| 2 | 1.0 |  | 0.80 |
| 3 | 1.0 |  | 0.80 |
| 4 | 0.90 |  | 1.0 |
| 5 | 0.90 |  | 0.80 |
| 6 | 1.0 |  | 0.80 |
| 7 | 1.0 |  | 1.0 |
| 8 | 1.0 |  | 0.80 |
| 9 | 1.0 |  | 0.80 |
| 10 | 0.90 |  | 0.80 |
| 11 | 1.0 |  | 1.0 |
| 12 | 0.90 |  | 0.80 |
| 13 | 1.0 |  | 0.80 |
| 14 | 1.0 |  | 1.0 |
