# Supplementary material for: Knowledge, attitude and practice of healthcare providers on mistreatment of women during labour and childbirth: A cross-sectional study in Tehran, Iran, 2021
Source: PLoS One. 2024 Oct 3;19(10):e0311346. doi: 10.1371/journal.pone.0311346 (PMC11449288; doi:10.1371/journal.pone.0311346)
Supplement: S1 Table — (DOCX) [file pone.0311346.s005.docx]

**S1 Table. Knowledge about mistreatment among maternity healthcare providers (n=255).**

| **Categories of mistreatment** | **Responses, n (%)** | | | |
| --- | --- | --- | --- | --- |
|  | **Yes** | **No** | **Do not know** |  |
| **Physical abuse** |  |  |  |  |
| Applying fundal pressure during delivery | 90 (35.3) | 155 (60.8) | 10 (3.9) |  |
| **Verbal abuse** |  |  |  |  |
| Using the phrase: “If you do not give birth, you will be taken to the operating room for a cesarean section” | 94 (36.9) | 150 (58.8) | 11 (4.3) |  |
| Using the phrase: “If you do not self-help, you will be responsible for the consequences” | 71 (27.8) | 175 (68.6) | 9 (3.5) |  |
| **Failure to meet professional standards of care** |  |  |  |  |
| Long waiting to receive services | 90 (35.3) | 156 (61.2) | 9 (3.5) |  |
| Frequent examinations for various reasons by MHCPs | 79 (30.0) | 172 (67.5) | 4 (1.6) |  |
| Refusal to provide pain relief | 149 (58.4) | 94 (36.9) | 12 (4.7) |  |
| **Poor rapport between women** **and providers** |  |  |  |  |
| Lack of respect for women’s preferred birth positions | 69 (27.1) | 177 (69.4) | 9 (3.5) |  |
| Denial of mobility | 114 (44.7) | 123 (48.2) | 18 (7.1) |  |
| **Health systems conditions and constraints** |  |  |  |  |
| Unreasonable requests of women by MHCPs | 215 (84.3) | 27 (10.6) | 13 (5.1) |  |
| Lack of privacy | 241 (94.5) | 14 (5.5) | 0 (0.0) |  |
| **Categories of mistreatment** | **Responses, n (%)** | | | |
|  | **Lack of supportive care** | **Neglect and abandonment** | **Discriminatory care** | **Do not know** |
| **Stigma and discrimination** |  |  |  |  |
| Discrimination based on medical conditions | 51 (20.0) | 22 (8.6) | 174 (68.2) | 8 (3.1) |

MHCPs: Maternity Healthcare Providers.
