# Supplementary material for: Knowledge, attitude and practice of healthcare providers on mistreatment of women during labour and childbirth: A cross-sectional study in Tehran, Iran, 2021
Source: PLoS One. 2024 Oct 3;19(10):e0311346. doi: 10.1371/journal.pone.0311346 (PMC11449288; doi:10.1371/journal.pone.0311346)
Supplement: S2 Table — (DOCX) [file pone.0311346.s006.docx]

**S2 Table. Attitudes about mistreatment among maternity healthcare providers (n=255).**

| **Categories of mistreatment** | **Responses, n (%)** | | | | |
| --- | --- | --- | --- | --- | --- |
|  | **Strongly agree** | **Agree** | **Uncertain** | **Disagree** | **Strongly disagree** |
| **Physical abuse** |  |  |  |  |  |
| Sometimes physical contact (slapping the thighs) can be used to encourage pregnant women to collaborate. | 1 (0.4) | 80 (31.4) | 24 (9.0) | 128 (50.2) | 23 (9.0) |
| Applying fundal pressure during birth | 3 (1.2) | 156 (61.2) | 24 (9.4) | 53 (20.8) | 19 (7.5) |
| **Verbal abuse** |  |  |  |  |  |
| Using threats to attract more collaboration in birth process | 2 (0.8) | 133 (52.2) | 24 (9.4) | 86 (33.7) | 10 (3.9) |
| Shouting at birthing woman | 1 (0.4) | 92 (36.1) | 28 (11.0) | 128 (50.2) | 6 (2.4) |
| **Failure to meet professional standards of care** |  |  |  |  |  |
| Informed consent process | 30 (11.8) | 192 (75.3) | 6 (2.4) | 24 (9.4) | 3 (1.2) |
| Neglect and abandonment | 3 (1.2) | 85 (33.3) | 31 (12.2) | 132 (51.8) | 4 (1.6) |
| **Poor rapport between women and providers** |  |  |  |  |  |
| Denial or lack of birth companions | 4 (1.6) | 54 (21.2) | 19 (7.5) | 157 (61.6) | 21 (8.2) |
| Denial of food and fluids | 0 (0.0) | 28 (11.0) | 14 (5.5) | 189 (74.1) | 24 (9.4) |
| Lack of accurate information about the progress of labour to companions | 0 (0.0) | 95 (37.3) | 23 (9.0) | 130 (51.0) | 7 (2.7) |
| **Health systems conditions and constraints** |  |  |  |  |  |
| Lack of privacy | 0 (0.0) | 38 (14.9) | 15 (5.9) | 182 (71.4) | 20 (7.8) |
| Staff restrictions on providing quality care to the mother | 13 (5.1) | 92 (36.1) | 18 (7.1) | 126 (49.4) | 6 (2.4) |
| **Stigma and discrimination** |  |  |  |  |  |
| Inability to provide equal services for all women during labour and delivery | 5 (2.0) | 132 (51.8) | 16 (6.3) | 95 (37.3) | 7 (2.7) |
| Discrimination based on sociodemographic characteristics | 5 (2.0) | 113 (44.3) | 16 (6.3) | 112 (43.9) | 9 (3.5) |
