# Supplementary material for: Knowledge, attitude and practice of healthcare providers on mistreatment of women during labour and childbirth: A cross-sectional study in Tehran, Iran, 2021
Source: PLoS One. 2024 Oct 3;19(10):e0311346. doi: 10.1371/journal.pone.0311346 (PMC11449288; doi:10.1371/journal.pone.0311346)
Supplement: S3 Table — (DOCX) [file pone.0311346.s007.docx]

**S3 Table. Practices about mistreatment among maternity healthcare providers (n=255).**

| **Categories of mistreatment** | **Responses, n (%)** | | | | |
| --- | --- | --- | --- | --- | --- |
|  | **Always** | **Often** | **Sometimes** | **Rarely** | **Never** |
| **Physical abuse** |  |  |  |  |  |
| I may slap labouring woman’s thighs to encourage her to collaborate during birth. | 0 (0.0) | 0 (0.0) | 29 (11.4) | 115 (45.1) | 111 (43.5) |
| Applying fundal pressure during delivery | 1 (0.4) | 15 (5.9) | 93 (36.5) | 84 (32.9) | 62 (24.3) |
| **Verbal abuse** |  |  |  |  |  |
| Shouting at the woman in case of non- collaboration | 3 (1.2) | 21 (8.2) | 123 (48.2) | 92 (36.1) | 16 (6.3) |
| **Failure to meet professional standards of care** |  |  |  |  |  |
| Keeping medical records and the results of tests and consultations confidential | 162 (63.5) | 68 (26.7) | 20 (7.8) | 3 (1.2) | 2 (0.8) |
| Providing information to woman about labour pain and how to relieve it | 94 (36.9) | 112 (43.9) | 38 (14.9) | 10 (3.9) | 1 (0.4) |
| Continuous or timely presence beside | 143 (56.1) | 60 (23.5) | 27 (10.6) | 20 (7.8) | 5 (2.0) |
| **Poor rapport between women and providers** |  |  |  |  |  |
| Warm welcoming at entrance to labour unit | 89 (34.9) | 121 (47.5) | 37 (14.5) | 7 (2.7) | 1 (0.4) |
| Introducing themselves (MHCPs) to woman | 85 (33.3) | 76 (29.8) | 55 (21.6) | 27 (10.6) | 12 (4.7) |
| Showing around maternity labour unit’s environment | 34 (13.3) | 54 (21.2) | 49 (19.2) | 74 (29.0) | 44 (17.3) |
| Establishing friendly communication | 79 (31.0) | 119 (46.7) | 45 (17.6) | 8 (3.1) | 4 (1.6) |
| **Health systems conditions and constraints** |  |  |  |  |  |
| Providing comfortable and calming environment | 36 (14.1) | 103 (40.4) | 74 (29.0) | 33 (12.9) | 9 (3.5) |
| Paying attention to safety in providing care and interventions | 150 (58.8) | 91 (35.7) | 11 (4.3) | 2 (0.8) | 1 (0.4) |
| Lack of privacy | 98 (38.4) | 104 (40.8) | 39 (15.3) | 10 (3.9) | 4 (1.6) |
| **Stigma and discrimination** |  |  |  |  |  |
| Respecting mothers and her companions’ beliefs and culture | 143 (56.1) | 98 (38.4) | 11 (4.3) | 2 (0.8) | 1 (0.4) |
