# Supplementary material for: Knowledge, attitude and practice of healthcare providers on mistreatment of women during labour and childbirth: A cross-sectional study in Tehran, Iran, 2021
Source: PLoS One. 2024 Oct 3;19(10):e0311346. doi: 10.1371/journal.pone.0311346 (PMC11449288; doi:10.1371/journal.pone.0311346)
Supplement: Attachment — Submitted filename: Response to Reviewers.docx [file pone.0311346.s009.docx]

TEHRAN UNIVERSITY OF MEDICAL SCIENCES

School of Public Health

November 4, 2023

**Dear Editor,**

I would like to express our sincere thanks for the detailed and constructive feedback and comments received on our paper. We have benefited a lot from those points and revised our manuscript based on the comments. Below, I would like to share our responses to the comments in the table. We hope that you will find the edited version with satisfaction.

Best regards,

Dr. Elham Shakibazadeh,

Corresponding author

| **No** | **Comments** | | **Responses** |
| --- | --- | --- | --- |
| **Editor** | | | |
| 1 | Please include a complete copy of PLOS’ questionnaire on inclusivity in global research in your revised manuscript. | We added the PLOS’ questionnaire on inclusivity in global research in the text.  Page 12; Lines: 235-237 | |
| 2 | We note that the grant information you provided in the ‘Funding Information’ and ‘Financial Disclosure’ sections do not match. When you resubmit, please ensure that you provide the correct grant numbers for the awards you received for your study in the ‘Funding Information’ section. | We revised and corrected the numbers. | |
| 3 | Please ensure that you include a title page within your main document. You should list all authors and all affiliations as per our author instructions and clearly indicate the corresponding author. | We edited the title page accordingly. | |
| 4 | Please include captions for your Supporting Information files at the end of your manuscript, and update any in-text citations to match accordingly. | We added captions for Supporting Information files at the end of the text.  Page 23; Lines: 419-425 | |
| 5 | There is need to indicate in the abstract that this is part of an implementation study. The document should also be well edited. | We added in the abstract that this was part of an implementation study. Also, we edited the document according to the reviewers' comments.  Page 2; Line: 25 | |
| **Reviewer 1** | | | |
| 1 | This topic was done previously in Iran with a well-developed scale, however the researcher design and use another scale! | The scale developed in Iran includes items about midwives’ knowledge and practice of respectful maternity care (RMC) and not the mistreatment of women during childbirth^1^. Evidence shows that RMC and mistreatment of women during childbirth are not the same concept, but occupy two extremes of a continuum. The absence or lessening of mistreatment during childbirth does not guarantee RMC for women and newborns during childbirth^2, 3^. To the best of our knowledge, there is no scale to examine maternity healthcare providers' (MHCPs) knowledge, attitudes, and practices regarding mistreatment during childbirth. Therefore, in this study, a self-report questionnaire prepared using the typology of mistreatment of women during childbirth by Bohren et al. and a literature review was used.  ^1^ Moridi M, Pazandeh F, Potrata B. Midwives’ knowledge and practice of Respectful Maternity Care: a survey from Iran. BMC Pregnancy and Childbirth, 2022; 22: 752.  ^2^ Shakibazadeh E, Namadian M, Bohren MA, Vogel JP, Rashidian A, Nogueira Pileggi V, et al. Respectful care during childbirth in health facilities globally: a qualitative evidence synthesis. BJOG, 2018; 125(8):932-42.  ^3^ Afulani PA, Diamond-Smith N, Phillips B, Singal S, Sudinharaset M. Validation of the person-centered maternity care scale in India. Reproductive Health, 2018; 15:147. | |
| 2 | The introduction section does not have accurate citations; it is recommended that all the sentence should be cited. Also, sentences and paragraphs have no conceptual connection. | Thank you for your recommendation. We rephrased the sentences and paragraphs for conceptual connection. We also added references to the sentences.  Page 3; Line: 59  Page 4; Lines: 67-74, Lines: 79-82, and Lines: 84-86 | |
| 3 | In the method's section, it is determined that they use random stratification; however, in the lines 145-146 showed that they did not select hospitals randomly. | We rephrased the sampling method.  Page 7; Line: 148  Page 8; Lines: 149-152 | |
| 4 | Also, the participants were not determined in this section. The MHCPs is not clear that refers to which kinds of providers? | In the “Setting and participants” sub-section, it is noted that MHCPs included obstetricians, midwives, obstetric residents, midwifery, and medical interns.  Page 6; Lines: 131-134 | |
| 5 | Another concern about the accuracy of this study is that how the scale was developed and is it suitable for all kind of health workers? | In this study, we first prepared a questionnaire using the typology of mistreatment of women during childbirth presented by Bohren et al. and a literature review. Next, we evaluated the validity and reliability of the scale. Face validity was assessed by five maternity healthcare providers (obstetricians, midwives, obstetric residents, midwifery, and medical interns) regarding the relevance, ambiguity, and difficulty of the questions, and minor changes were made to the items. Content validity was assessed by calculating the content validity index (CVI) and content validity ratio (CVR) by an expert panel (n = 10) in the fields of midwifery, reproductive health, obstetrics, health education and promotion, and health services management. To determine reliability, the questionnaire was administered to 30 maternity healthcare providers twice at intervals of two weeks, and the intraclass correlation coefficient (ICC) and Cronbach's alpha coefficient were calculated.  Page 8; Lines: 163-171  Page 9; Lines: 172-194 | |
| 6 | The scale's items are not completely consistent with the content extracted in table 1. | Table 1 provides information about the study hospitals. While the scale's items include categories of mistreatment during childbirth (physical abuse, verbal abuse, stigma and discrimination, failure to meet professional standards of care, poor rapport between women and providers, and health systems conditions and constraints) (Table 2). | |
| 7 | All findings were not discussed appropriately. | We made some elaborations in the discussion section.  Page 19; Lines: 318-320  Page 20; Lines: 356-359 | |
| 8 | Table 7 is not clear and may be confusing for the audiences. | Table 7 (which is currently referred to as Table 4 in the paper) shows the association of sociodemographic characteristics and knowledge, attitude, and practice scores of the participants. We summarized the description of this table, and referred readers to the table.  Page 15; Lines: 282-302  Page 16; Lines: 303-307 | |
| 9 | The balance between the numbers of words in different sections of the manuscript is not observed. | We have summarized the results section by moving some tables to the Supporting information section. | |
| **Reviewer 2** | | | |
| 1 | This paper was well written with clear aims, but a few strong rationales underlying the importance of the topic. In addition, some problems should be addressed to improve the manuscript. | We sincerely appreciate your feedback. We rephrased the sentences and paragraphs to mention the importance of the issue in paragraphs 4 and 5.  Page 5; Lines: 92-102 and Lines: 103-115 | |
| 2 | While the paper build upon a KAP of providers related to mistreatment of women during childbirth, it is not provided a sufficient and specific information about Exams & Procedures: Informed consent, exposed (vaginal exams, …), confidential information. These items are very important indexes in RMC. Also, it is better to describe an unsupportive birth environment items such pain relief, neglect and also clean up blood …. | In the questionnaire (S1 Appendix), item 7 of knowledge related to “vaginal exams”, item 5 of attitude related to “informed consent”, and item 6 of practice related to “confidentiality of information”. “Pain relief” was also mentioned in item 9 of knowledge and item 8 of practice, and “neglect, abandonment, or long delays” were mentioned in item 3 of knowledge, item 8 of attitude, and item 9 of practice. | |
| 3 | This manuscript must contain Ethics approval and consent to participate, funding and acknowledgement. It is better to give permission from hospitals to mention the names of hospitals in this article. | Ethical approval and consent to participate, and acknowledgement in the text are mentioned. As required by the journal, we entered the Funding Information section in the Financial Disclosure section of the Submission System. Permission to mention the names of the hospitals was also obtained in this paper, and we added them in the text.  Page 11; Lines: 227-229  Page 12; Lines: 230-234  Page 23; Lines: 426 and Page 24; Lines:  427-429 | |
| **Reviewer 3** | | | |
| 1 | The word “Practice” used in the title seems not to match with the outcome. Knowledge and attitude of mistreatment of women during labour and childbirth is in line. | In our study, practices scale was assessed using 14 items, and the results showed that only 3.1% of the participants reported moderate mistreatment practices towards birthing women. However, like many other cross-sectional studies, we assessed the practices in our study using self-report scale, which may have caused bias owing to the sensitivity of the mistreatment of women during childbirth. We attempted to reduce this bias by reassuring the participants’ anonymity and confidentiality. However, assessing the practices of MHCPs in an observational study will also be important.  We added this to the study limitations section.  Page 22; Lines: 401-406 | |
| 2 | “Most participants (82.7%) had poor knowledge about physical abuse, verbal abuse, poor rapport between women and providers, and failure to meet professional standards of care”. 82.7% is not “most” authours should consider using “majority”) Is the 82.7% poor knowledge relating each of the items listed; verbal abuse, poor rapport between? etc | We rephrased the sentence for better comprehension.  Page 2; Lines: 33-38 | |
| 3 | “Self-reported practices of different types of mistreatment were not common and only 3.1% of the participants were in moderate level”. This statement is not clear, authors could consider rephrasing | We rephrased the sentence for better comprehension.  Page 2; Lines: 38-40 | |
| 4 | “These interventions should include designing and implementing continuing education courses and revising the educational curriculums to increase knowledge, strengthen positive attitudes, modify the practice of maternity healthcare providers, overcoming staff shortages, paying staff fairly, establish a support culture for mother-centered and respectful care, and increase the quality of maternity care”. This statement is long to follow. Kindly revise. Authors could consider ending at health care providers; then continue other interventions may include...... | Thank you for pointing this out. We revised the statement accordingly.  Page 3; Line: 48 and Line: 50 | |
| 5 | Line 79.... However, reports of disrespectful maternity care are on the rise worldwide [10-12]. Can this be further explained? Why the rise? | We rephrased this sentence in the text.  Page 4; Lines: 84-86 | |
| 6 | “In a study of 38 hospitals and health centers in Ethiopia, 74% of women reported experiencing some forms of mistreatment [13]. A systematic review of five studies in Ethiopia, Kenya, Nigeria and Tanzania found that the prevalence of D&A ranged from 15 to 98% [14]. A WHO-led study (2019) in Ghana, Guinea, Nigeria and Myanmar found that 35.4% of women experienced physical or verbal abuse, or stigma or discrimination during childbirth [15]”. What reasons why attributed to prevalence in these referenced studies? | We edited some sentences in the Introduction section. Reference to the prevalence in these studies is provided to further explain the sentence (.... growing evidence shows that mistreatment during childbirth has become a common experience for women worldwide [12-15]).  Page 4; Lines: 84-91 | |
| 7 | “A main step in designing interventions to prevent mistreatment is to assess the level of knowledge, attitudes and practices (KAP) of MHCPs, with a goal to improve negative behaviors and reinforce positive behaviors”. Is this statement author's idea? If not, kindly reference. | Yes; this statement is the author's idea. | |
| 8 | The parameters used in the sample size calculation are not clear. e.g. mean KAP is questionable. Mean knowledge of course can be determined but combined KAP mean, I don't think so. Authors should provide further explanation and should kindly provide reference of the mean and SD values. Again in the abstract, author's stated total enumeration, how is this related to the sample size calculation. This will need further elaboration. | We provided further explanations for the parameters used in the sample size calculation, and added references to the mean and SD values. We also edited the results section in the abstract.  Page 7; Lines: 143-147  Page 2; Line: 33 | |
| 9 | Line 143: “The sampling method was random stratification” Is this for participants or sites? | We rephrased the sampling method for better comprehension.  Page 7; Line: 148  Page 8; Lines: 149-152 | |
| 10 | Line 194 “Some items had reverse scoring”. Which of the items had these? | We added it in the text.  Page 11; Lines: 206-207 | |
| 11 | Was the score categorization for attitudes also based on Bloom's cut-off point? Otherwise this should be referenced. | Yes; the score categorization for knowledge, attitudes, and practices was based on Bloom's cut-off point.  We rephrased the sentence for better comprehension.  Page 10; Lines: 199-201 | |
| 12 | “Due to abnormality of knowledge and practice scores (p = 0.01 and p = 0.04, respectively), we used Mann-Whitney U and Kruskal-Wallis tests”. What was these tests specifically used for, apart from the mere mentioning of abnormality? It serve readers well if detailed analysis is presented, as it stands, it looks scanty | We revised the statistical analysis.  Page 11; Lines: 219-224 | |
| 13 | Line 219..... 33.8% is not “most”. Authors should kindly me mindful of these terms, most...majority etc. same applies here “Most of them had permanent job (n=54; 21.2%).” | Thank you for pointing this out. We revised the sentences accordingly.  Page 12; Lines: 242-244 | |
| 14 | Was knowledge, practice and attitude scores expressed in percentage? Authors should provide readers with detailed explanation. | We added explanations about this in the Methods section (Scoring and Statistical analysis).  Page 11; Lines: 211-215 | |
| 15 | MHCPs attitudes regarding mistreatment: Results here are presented in freq (%). Authors failed to write about this in the analysis (summary statistics was used to present demographic information, mentioned in the analysis). Same applies to practice...kindly check these and revise. | We revised the statistical analysis.  Page 11; Lines: 217-225 | |
| 16 | Table 6 and 7 seem to be presenting the same information. Authors could stick to one. Again, description of table 7 could be summarized with significant predictor variables. No need to present all details as it makes reading boring ... kindly present few and refer readers to the table. | Table 6 shows the practices of providers regarding mistreatment of women during labour and childbirth, and Table 7 (which is currently listed as Table 4 within the paper) shows the association of sociodemographic characteristics and knowledge, attitude, and practice scores of providers. We summarized the description of this table, and refer readers to the table.  Page 15; Lines: 282-302  Page 16; Lines: 303-307 | |
