# Supplementary material for: Knowledge, attitude and practice of healthcare providers on mistreatment of women during labour and childbirth: A cross-sectional study in Tehran, Iran, 2021
Source: PLoS One. 2024 Oct 3;19(10):e0311346. doi: 10.1371/journal.pone.0311346 (PMC11449288; doi:10.1371/journal.pone.0311346)
Supplement: Attachment — Submitted filename: Response to Reviewers.docx [file pone.0311346.s010.docx]

TEHRAN UNIVERSITY OF MEDICAL SCIENCES

School of Public Health

June 15, 2024

**Dear Editor,**

I would like to express our sincere thanks for the detailed and constructive feedback and comments received on our paper. We have benefited a lot from those points and revised our manuscript based on the comments. Below, I would like to share our responses to the comments in the table. We hope that you will find the edited version with satisfaction.

Best regards,

Dr. Elham Shakibazadeh,

Corresponding author

| **No** | **Comments** | | **Responses** |
| --- | --- | --- | --- |
| **The reviewer’s comments** | | | |
| 1 | In the abstract, line 34: Most participants (82.7%) had poor knowledge about physical abuse, verbal abuse, poor rapport between women 35 and providers, and failure to meet professional standards of care. The use of “most” seems inappropriate, I will suggest “majority” instead of “most”. Does the 82.7% poor knowledge refer to each of the domains “physical abuse, verbal abuse, poor rapport between women and providers, and failure to meet professional standards of care”. This is not clear. | We rephrased the sentence for better comprehension.  Page 2; Lines: 33-36 | |
| 2 | In the abstract, line 28: All maternity healthcare providers (obstetricians, midwives) and students were invited to the study… “All eligible maternity healthcare providers (obstetricians, midwives) and students were invited to the study”. | Thank you for pointing this out. We revised the statement accordingly.  Page 2; Line: 26-28 | |
| 3 | In the abstract, line 36: Most participants (69.4%) were 36 alright with physical abuse, verbal abuse, and discrimination. This statement is not also clear. What does authors mean by “alright”. | We rephrased the sentence for better comprehension.  Page 2; Lines: 37-38 | |
| 4 | In the abstract, conclusion: The conclusion seem too long, authors should revise and go straight to conclude study findings. | We summarized the conclusion.  Page 3; Lines: 44-48 | |
| 5 | In the introduction, there are no information on “knowledge and practices of mistreatment of healthcare providers”. A Brief information on the this main outcome will be important in the background. | We provided the information in page 5, lines 99-108. | |
| 6 | In the methods, line 139-142: “Considering that the mean scores of KAP of participants were calculated on the basis of 100, the standard deviation of the score was 16.7 and the number of samples was determined with 95% confidence level (Z1- α 2 = 1.96) and a maximum error of two scores estimated this mean” how did authors come by the values used in the calculation of sample size?. If this is a previous study, provide reference. | We provided further explanations for the parameters used in the sample size calculation, and added references to the mean and SD values.  Page 7; Lines: 139-144  Page 11; Lines: 209-211 | |
| 7 | “Due to normal attitude scores (p = 0.17), we used independent samples t-test and one-way (ANOVA)” what exactly was this used for? Authors should expand on this. | We revised the statistical analysis.  Page 11; Lines: 217-222 | |
| 8 | Which people were involved in the data collection, how were they trained? | We added explanations about this in the Data collection section.  Page 8; Lines: 151-152  Page 8; Lines: 153-156 | |
| 9 | Line 212- 213, “This study did not require the respondents to identify themselves and for confidentiality, ID was used instead of names for the questionnaires.” This information may not be necessary. | We removed the sentence.  Page 12; Line: 299 | |
| 10 | In the results, line 219: 33.8% is not most, this may need revision. | Thank you for pointing this out. We revised the sentence accordingly.  Page 12; Lines: 238-240 | |
| 11 | The use of “most”, “majority” need to be used meticulously in the entire work. I suggest authors consider when appropriate to use these descriptive words. | We revised the use of these terms in the entire text.  Page 2; Lines: 33-36  Page 2; Lines: 37-38  Page 12; Lines: 238-240 | |
| 12 | Line 244: the scoring is explained “in” the Methods section | We added it in the text.  Page 13; Line: 248 | |
