# Supplementary material for: Knowledge, attitude and practice of healthcare providers on mistreatment of women during labour and childbirth: A cross-sectional study in Tehran, Iran, 2021
Source: PLoS One. 2024 Oct 3;19(10):e0311346. doi: 10.1371/journal.pone.0311346 (PMC11449288; doi:10.1371/journal.pone.0311346)
Supplement: Attachment — Submitted filename: Response to Reviewers.docx [file pone.0311346.s011.docx]

TEHRAN UNIVERSITY OF MEDICAL SCIENCES

School of Public Health

August 28, 2024

**Dear Editor,**

I would like to express our sincere thanks for the detailed and constructive feedback and comments received on our paper. Below, I would like to share our responses to the comments in the table. We hope that you will find the edited version with satisfaction.

Best regards,

Dr. Elham Shakibazadeh,

Corresponding author

| **No** | **Comments** | | **Responses** |
| --- | --- | --- | --- |
| **The reviewer’s comments** | | | |
| 1 | In the data collection section, from lines 151 to 156 written in one sentence, write in several sentences | Thank you for pointing this out. We revised the statement accordingly.  Page 7; Lines: 144-149 | |
| 2 | It is better to use univariate and multivariate regression in the analysis | We included univariate and multiple regression in the analysis.  Page 11; Lines: 213-220  Page 18; Lines: 294-315  Page 19; Lines: 316-318 | |
